# Effect of sevoflurane on the inflammatory response during cardiopulmonary bypass in cardiac surgery: the study protocol for a randomized controlled trial

**DOI:** 10.1186/s13063-020-04809-x

**Published:** 2021-01-06

**Authors:** Thiago Augusto Azevedo Maranhão Cardoso, Gudrun Kunst, Caetano Nigro Neto, José de Ribamar Costa Júnior, Carlos Gustavo Santos Silva, Gisele Medeiros Bastos, Jéssica Bassani Borges, Mario Hiroyuki Hirata

**Affiliations:** 1 Department of Surgery and Anesthesia, Dante Pazzanese Institute of Cardiology, Captain Pinto Ferreira Street, 62, ap 92, Jardim Paulista, São Paulo, 01423-020 Brazil; 2 Department of Anaesthetics, Intensive Care Medicine and Pain Therapy, King’s College Hospital NHS Foundation Trust, Denmark Hill, London, UK; 3 Department of Interventional Cardiology, Dante Pazzanese Institute of Cardiology, São Paulo, Brazil; 4 Molecular Cardiology Research Laboratory, Dante Pazzanese Institute of Cardiology, São Paulo, Brazil; 5 Department of Clinical and Toxicological Analysis, School of Pharmaceutical Sciences, University of São Paulo, São Paulo, Brazil

**Keywords:** Volatile anesthetics, Cardiac anesthesia, Cardiac surgery, Systemic inflammatory response, Cardiopulmonary bypass

## Abstract

**Background:**

Recent experimental evidence shows that sevoflurane can reduce the inflammatory response during cardiac surgery with cardiopulmonary bypass. However, this observation so far has not been assessed in an adequately powered randomized controlled trial.

**Methods:**

We plan to include one hundred patients undergoing elective coronary artery bypass graft with cardiopulmonary bypass who will be randomized to receive either volatile anesthetics during cardiopulmonary bypass or total intravenous anesthesia. The primary endpoint of the study is to assess the inflammatory response during cardiopulmonary bypass by measuring PMN-elastase serum levels. Secondary endpoints include serum levels of other pro-inflammatory markers (IL-1β, IL-6, IL-8, TNFα), anti-inflammatory cytokines (TGFβ and IL-10), and microRNA expression in peripheral blood to achieve possible epigenetic mechanisms in this process. In addition clinical endpoints such as presence of major complications in the postoperative period and length of hospital and intensive care unit stay will be assessed.

**Discussion:**

The trial may determine whether adding volatile anesthetic during cardiopulmonary bypass will attenuate the inflammatory response.

**Trial registration:**

ClinicalTrials.gov NCT02672345. Registered on February 2016 and updated on June 2020.

**Supplementary Information:**

The online version contains supplementary material available at 10.1186/s13063-020-04809-x.

## Introduction

### Background and rationale

Since 1953, when Gibbon performed the first successful human intracardiac operation using a mechanical extracorporeal pump oxygenator [1], cardiac surgery with cardiopulmonary bypass (CPB) has been transformed into a relatively standardized medical procedure, performed on millions of patients around the world.

Despite major improvements during the last decades, CPB continues to be associated with an undesirable inflammatory reaction affecting the brain, kidneys, liver, lungs, and heart. These inflammatory reactions are the result of contact of patients’ blood with the non-biocompatible circuit of the CPB machine. This promotes activation of leukocytes and subsequently activation of inflammatory proteins, such as polymorphonuclear-elastase (PMN-elastase) and cytokines such as interleukins (IL-1β, IL-6, IL-8) and alpha tumor necrosis factor (TNFα) [2].

There are a few underpowered clinical studies evaluating the effect of sevoflurane on the inflammatory response during CPB [3–6]. Based on these proof-of-concept studies, it is hypothesized that sevoflurane contributes to the protection of target organs, due to intracellular signal transduction modulations reducing ischemia reperfusion injury (IRI), which occurs during CPB [7]. However, the exact mechanisms involved in these anti-inflammatory effects have not been elucidated [2].

Recently, an experimental study showed that the activation of neutrophil granulocytes during CPB was inhibited by sevoflurane [2]. The authors concluded that during ex vivo normothermic CPB, the inflammatory response was reduced by decreasing Mac-1 expression and reducing PMN-elastase release. In order to further validate these findings, we designed this clinical trial.

Some studies suggest a possible contribution of sevoflurane to the modification of microRNA (miRNAs) expression involved in the control of cytokine synthesis and release, among the molecules involved in the inflammatory response during the IRI process related to CPB [8].

The main objective of this study is to determine if sevoflurane can reduce serum levels of PMN-elastase if given during CPB in elective of coronary artery bypass graft surgery when comparing with total intravenous anesthesia (TIVA).

Secondary endpoints will include serum concentrations of other pro-inflammatory markers such as interleukins IL-1β, IL-6, IL-8, TNFα, and anti-inflammatory cytokines such as TGFβ and IL-10, assessing epigenetic mechanisms [9, 10]. Furthermore, clinical secondary endpoints include time to extubation, length of hospital and intensive care unit (ICU) stay, and major complications in the immediate postoperative period.

### Objectives

We hypothesized that sevoflurane can reduce the CPB induced inflammatory response by reducing serum PMN-elastase levels in patients undergoing coronary artery bypass graft (CABG) surgery with CPB.

### Trial design

A parallel group, randomized controlled, single-blinded single-center trial with 1:1 allocation ratio.

## Methods: participants, interventions, and outcomes

### Study setting

The study setting is based on a hospital specialized in cardiovascular diseases localized in the city of São Paulo in Brazil and where all data collection and analysis will be done.

### Eligibility criteria

We will enroll 100 patients undergoing elective CABG surgery at the Dante Pazzanese de Cardiology Institute São Paulo/Brazil. Exclusion criteria are as follows: pregnancy, preexisting diseases such as asthma, chronic obstructive pulmonary disease (COPD), and/or autoimmune diseases; planned combined interventions (e.g., CABG plus valve surgery); body mass index (BMI) > 40 kg/m^2^; unstable or ongoing angina, recent (less than 1 month) or ongoing acute myocardial infarction; use of corticosteroids, anti-inflammatory, and/or immunosuppressive medications that cannot be discontinued at least 24 h of surgery; previous unusual response to an anesthetic agent; inclusion in other randomized controlled studies in the previous 30 days; current clinical history of decompensated heart and hepatic and/or kidney failure (Table 1).

**Table 1:** Inclusion and exclusion criteria

Inclusion criteria	Exclusion criteria
Age > 18 years	Patients receiving corticosteroids, anti-inflammatory, and/or immunosuppressive medications
Written informed consent	Reoperation of cardiac surgery
Scheduled procedure	Asthma carrier, COPD, autoimmune diseases
Cardiac surgery with CPB of coronary artery bypass graft	Inclusion in other randomized controlled studies
	Emergency operation
	Unstable angina
	Pregnancy
	Acute myocardial infarction (< 1 month)
	Morbid obesity (BMI > 40 kg/m^2^)
	Cardiac, hepatic, and/or renal decompensated insufficiency clinically proven

### Who will take informed consent?

Participants will be older than 18 years old who will sign the written informed consent whose collection will be made by the anesthetist responsible for the procedure according to the local ethics committee board approval.

### Additional consent provisions for collection and use of participant data and biological specimens

If an auxiliary study involving the same patient population as this study, due to the blood samples that will be stored, another consent form must be signed.

## Interventions

### Explanation for the choice of comparators

Participants will be randomized in two groups: sevoflurane (SEVO) and total intravenous anesthesia (TIVA) with a 1:1 allocation as per a computer-generated randomization schedule.

### Intervention description

The SEVO group will receive sevoflurane in addition to intravenous agents according to local protocol and expertise. Sevoflurane will be administered only during CPB with at least 0.7 of minimum alveolar concentration (MAC) not exceeding the maximum value of 1.5 MAC.

The TIVA group will receive intravenous agent (midazolam, fentanyl, or sufentanyl) and no sevoflurane. Agents for TIVA will be administered as both target-controlled infusion (TCI) and manually controlled infusion (MCI) according to local protocol and expertise.

All participants will receive perioperative supportive treatments according to their institutional practice, including general anesthesia; pacing, inotropic drugs; mechanical ventilation; postoperative sedation/analgesia; diuretics; intravenous fluids; antibiotics and invasive monitoring including, but not limited to, invasive arterial pressure; electrocardiogram; central venous pressure; pulse oximetry; temperature; urine output; arterial blood gases; and frequent routine laboratory examinations.

### Criteria for discontinuing or modifying allocated interventions

In those situations, in which the individual meets the eligibility criteria and is therefore selected for the study protocol, they may be discontinued or modified, those cases in which the CABG surgical schedule was changed during the intraoperative period or when the individual dies from the intervention in the first 24 h of surgery.

### Strategies to improve adherence to interventions

- Daily guidance on the importance of following the study guidelines by the anesthetists assigned to perform the anesthesia of the selected patients for better adherence.
- Instructions to perfusionists on how to use the sevoflurane vaporizer within the limits recommended by the study protocol; ensure that the vaporizer is filled with sevoflurane; start the use of sevoflurane in cases randomized to the SEVO group since the beginning of CPB.
- Notification that there will be a check of the medical record every day to check the information regarding secondary objectives.
- Reinforce that patients selected for the SEVO group will receive the sevoflurane anesthetic only during CPB.
- Importance of calling patients 1 month after surgery to check their vitality.

### Relevant concomitant care permitted or prohibited during the trial

The main relevant concomitant care and intervention prohibited during the study period is the fact that the anesthesiologist responsible for the procedure does not use sevoflurane during the pre- and post-CPB period.

### Provisions for post-trial care

There is no anticipated harm and compensation for trial participation.

### Outcomes

Primary endpoint of the study is to determine whether sevoflurane used during CPB can reduce PMN-elastase levels 90 min after the onset of CPB.

Secondary endpoints will include the following: dosage of the other pro-inflammatory markers such as interleukins IL-1β, IL-6, IL-8, and TNFα and anti-inflammatory cytokines such as beta tumor growth factor (TGFβ) and IL-10, to analyze epigenetic mechanisms such as miRNA that can elucidate the anti-inflammatory mechanism of volatile anesthetics, time to extubation, length of hospital and ICU stay, and major complications throughout the hospitalization period up to 30 days after the surgery. We also collect the number of postoperative complications: need for pacemaker, reoperation, reintubation, arrhythmias, massive transfusion, myocardial infarction, acute kidney failure, death. Definitions of the outcomes are presented in the Additional file 1. To perform this, after 30-day telephone contact (patient and/or relatives) will be used. In case of telephone follow-up loss, the following methods will be used: contact the patient’s general practitioner, contact the city municipality, and send a letter to the home address of the patient.

The data will be inserted by the responsible researcher in a database based on a paper sheet approved by the ethics committee and filled with the relevant information from the pre-, intra-, and postoperative period. Information on laboratory dosages will also be entered by the responsible researcher after the transmission of these data by the molecular biology laboratory responsible for the samples.

Data will be stored in an electronic database with no patient identifiers (a numeric code will be used).

### Participant timeline

**Table:** 

	Study period						
	Enrolment	Allocation	Post-allocation				Close-out
TIMEPOINT**	***− t***_***1***_	**0**	***T***_***0***_	***T***_***1***_	***T***_***2***_	***T***_***3***_	***t***_***x***_
**ENROLMENT:**							
**Eligibility screen**	X						
**Informed consent**	X						
**Allocation**		X					
**INTERVENTIONS:**							
***[SEVO group]***							
***[TIVA group]***							
**ASSESSMENTS:**							
***[age, sex, weight, height, BMI, ASA, NYHA, risk factors, preoperative medications]***		X					
***[SEVO (%), gas flow (L/min), CPB flow (l/min/m***^***2***^***), BIS, Temperature (°C), MAP (mmHg), Ht, Hb, Ph, Lac, Glic, Na, K]***			X	X	X	X	
***[PMN-elastase, IL-1β, IL-8, TGFβ, IL-6, TNFα, IL-10]***							X
***[Postoperative complications]***							X

*SEVO* sevoflurane, *TIVA* total intravenous anesthesia, *BMI* body mass index, *ASA* American Society Of Anesthesiology (Physical State), *NYHA* New York Heart Association (Functional Class), *Cpb* cardiopulmonary bypass, *BIS* bispectral index, *MAP* mean arterial pressure, *Ht* hematocrit, *Hb* hemoglobin, *Ph* hydrogen potential, *Lac* lactate, *Glic* blood glucose, *Na* sodium, *K* potassium, *PMN* polymorphonuclear, *IL* interleukin, *TGF* transforming growth factor, *TNF* tumor necrosis factor

### Sample size

The sample size is based on the calculations performed from the data provided by the authors of an experimental article [2] whose difference in nanograms per milliliter is greater than 50% between the means of the groups that used or not the volatile anesthetic sevoflurane during the longest CPB applied by those authors (90 min) with a standard deviation (SD) of 30.71, a significance level of 5%, and a power of the sample of 80% generated from 100 patients causing a significant reduction in plasma PMN-elastase levels in the group that included sevoflurane in the gas circuit. Selected patients who die during the entire follow-up will be cited at the end of the trial and will remain in the study for a final analysis of the results. Therefore, the sample size calculation was consistent with the proposal to assess the inflammatory response by measuring PMN-elastase 90 min after the onset of CPB. We use the SPSS V.19.0 software.

The analysis of subgroups of this study will be based on the cause and effect relationship between producing a reduction in the inflammatory response after 90 min of exposure to sevoflurane anesthetic during CPB and the intubation times, length of stay in the ICU, and length of hospital stay.

Finally, loss of follow-up due to intraoperative death was not included, as the death rate in this period is very low for this type of procedure at the Dante Pazzanese Institute of Cardiology.

### Recruitment

Recruitment will be carried out actively by the responsible researcher among those patients evaluated by the cardiology and cardiac surgery team with indication for CABG surgery.

### Assignment of interventions: allocation

#### Sequence generation

The participants will be allocated with centrally provided sealed opaque envelopes with the use of a permuted-block design stratified. We will use randomization blocks of 20. Patients will be unaware of the group assignments.

#### Concealment mechanism

Participants will be randomized using TENALEA, which is an online, central randomization service. Allocation concealment will be ensured, as the service will not release the randomization code until the patient has been recruited into the trial, which takes place after all criteria of inclusion and exclusion have been met.

#### Implementation

Anesthesiologists will provide the trial treatment intervention and, consequently, will know patients’ group allocation but they will be blinded to the postoperative medication prescription, data collection, data entry, or data analysis. Investigators and clinical personnel caring for patients, including intensive care physicians, will be blinded to the study drug for the duration of the trial. The data entered in the form will be held by the responsible researcher.

### Assignment of interventions: blinding

#### Who will be blinded

Due to the nature of the intervention, only participants will be blinded to the allocation. A laboratory worker will be responsible for feeding the data to the computer on separate data sheets and providing a final spreadsheet ready for statistical analysis.

#### Procedure for unblinding if needed

There is no procedure for unblinding.

### Data collection and management

#### Plans for assessment and collection of outcomes

**Fig. 1:**
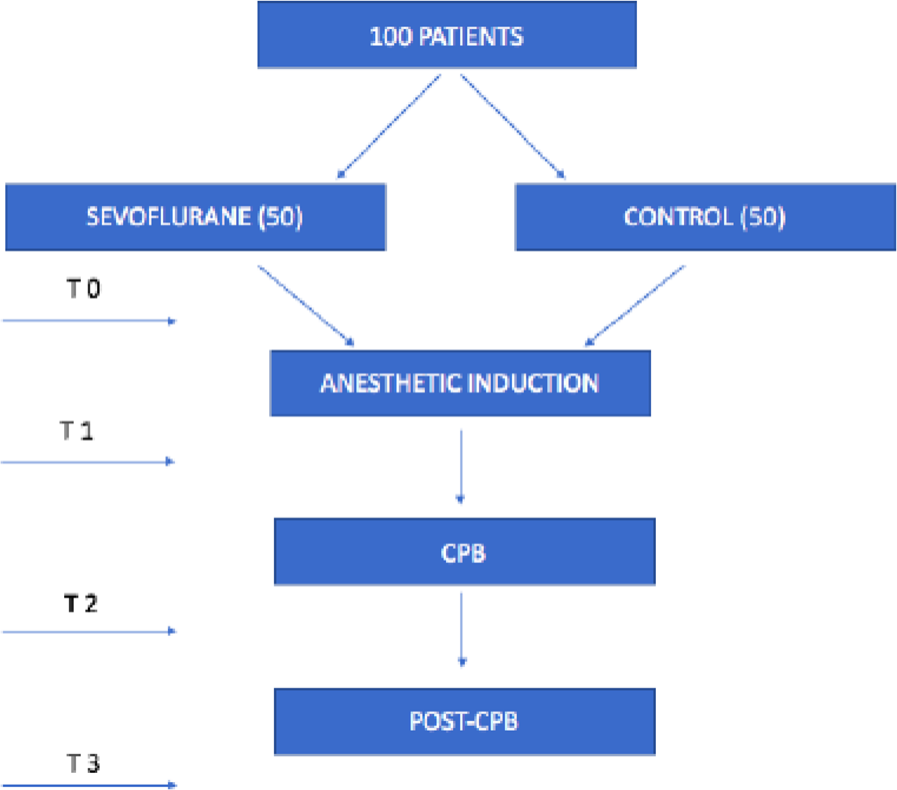
Flow chart about blood collection

In order to analyze messenger RNA (mRNA) and miRNA expression, DNA methylation, and inflammatory protein concentrations, whole blood will be collected from SEVO and TIVA groups at four different time points: before the anesthetic induction (T0), just before the start of the CPB (T1), 90 min post-start of the CPB (T2), and 24 h post-start of the CPB (T3) (Fig. 1).

The participant will be excluded from the study if sevoflurane is used at any other time during the intraoperative period other than during CPB and if a different type of surgical intervention other than the proposed myocardial revascularization occurs or patient refusal at any time. Intensive care will not be controlled, and the procedures and medications performed in the ICU will follow the protocols of that institution.

Data will be collected at the start of surgery, at the discharge from the ICU discharge, and at hospital discharge from the data collection forms. We will record perioperative variables such as gas flow, CPB flow, bispectral index value (BIS), temperature, mean arterial pressure (MAP), and blood gas values such as lactate, pH, hematocrit, hemoglobin, sodium, and potassium. Surgical characteristics including CPB duration and aortic cross-clamping duration will also be collected. With regard to volatile anesthetic use, the vaporizer dial should remain in the 1.5 to 3% MAC position.

The 30 days follow-up will focus on the major complications (e.g., vasoplegia, congestive heart failure, massive blood loss and transfusions, acute myocardial infarction, and death), hospital readmissions, and survival.

#### Plans to promote participant retention and complete follow-up

In case of telephone follow-up loss, the following methods will be used: contact the patient’s general practitioner, contact the city municipality, and send a letter to the home address of the patient.

### Data management

The data will be inserted by the responsible researcher in a database based on a paper sheet approved by the ethics committee and filled with the relevant information from the pre-, intra-, and postoperative period. Information on laboratory dosages will also be entered by the responsible researcher after the transmission of these data by the molecular biology laboratory responsible for the samples.

### Data will be stored in an electronic database with no patient identifiers (a numeric code will be used)

#### Confidentiality

Data will be stored in an electronic database with no patient identifiers (a numeric code will be used). Will participants be allocated an individual trial identification number and will participant’s details will be stored on a secure database. Only members of the study’s Data Analysis Commission will be entitled to handle the data. Will anonymized trial data be shared with other researchers to enable international prospective meta-analyses.

### Plans for collection, laboratory evaluation, and storage of biological specimens for genetic or molecular analysis in this trial/future use

#### Laboratory dosages

##### RNA isolation

Whole blood from patients will be collected into commercially available EDTA-treated tubes, and total RNA (including miRNA) will be isolated using TRIzol™ LS Reagent (Invitrogen) according to the manufacturer’s instructions. RNA quantification and integrity will be determined by Qubit® 2.0 Fluorometer (Life Technologies) and Agilent 2200 Tape Station® platform (Agilent Technologies), respectively.

##### Gene expression

For cDNA synthesis, 800 ng of total RNA and High-Capacity cDNA Reverse Transcription kit (Life Technologies) will be used following the manufacturer’s instructions. mRNA quantification of *IL-1B*, *IL-6*, *IL-8*, *IL-10*, *IL-4*, *TNFα*, and *TGFβ1* e *ELANE* will be performed by quantitative RT-PCR (qPCR) using TaqMan® Gene Expression Assay (Life Technologies) and Rotor-Gene® platform (Qiagen). The *geNorm* software (Primer Design Ltd.) will be used for selection of the most stable endogenous reference between glyceraldehyde 3-phosphate dehydrogenase (*GAPDH*), beta-2-microglobulin (*B2M*), hypoxanthine phosphoribosyltransferase 1 (*HPRT1*), succinate dehydrogenase complex flavoprotein subunit A (*SDHA*), ubiquitin C (*UBC*), and hydroxymethylbilane synthase (*HMBS*).

PCR will be performed in triplicate and in a multiplex format using Quantifast® Multiplex PCR assay (Qiagen). Thus, target genes and endogenous reference will be labeled with fluorescent dye FAM and VIC, respectively. Data will be analyzed using Rotor-Gene® Q - Pure detection software (Qiagen). The results will be obtained by the relative quantification method (2^−ΔΔCt^) [11].

##### miRNA expression

miRNA analysis will be performed by qPCR. For this, the software Ingenuity® Pathway Analysis (IPA®) (Qiagen) will be used to select about ten miRNA which can regulate the genes differently expressed between the study groups. Thus, cDNA synthesis and PCR will be performed using TaqMan® MicroRNA Reverse Transcription Kit (Applied Biosystems™), TaqMan® MicroRNA Assays (Applied Biosystems™), and TaqMan® Universal PCR Master Mix (Applied Biosystems™). Data will be analyzed using Rotor-Gene® Q - Pure detection software (Qiagen). The results will be obtained by the relative quantification method (2^−ΔΔCt^) [11].

##### DNA methylation

DNA methylation at specific CpG sites in *IL-1B*, *IL-6*, *IL-8*, *IL-10*, *IL-4*, *TNFα*, and *TGFβ1* e *ELANE* will be analyzed by pyrosequencing using PyroMark Q24 platform (Qiagen). For this, Genomic DNA will be purified from blood leucocytes by QIAmp DNA Blood Maxi Kit (Qiagen) according to the manufacturer’s instructions. The DNA quantification and purity will be analyzed by Qubit® 2.0 Fluorometer (Invitrogen) and Nanodrop ND-1000 (NanoDrop Technologies), respectively. The analyses of DNA methylation will be performed by bisulfite conversion using the EpiTect Fast DNA Bisulfite Conversion Kit (Qiagen) followed by DNA amplification using PyroMark PCR Kit (Qiagen) and PyroMark CpG Assays (Qiagen). Before the sequencing reaction, PCR product will be purified and denatured using the PyroMark Vacuum Workstation (Qiagen).

##### Protein measurements

IL-1β, IL-6, IL-8, IL-10, IL-4, TNFα, and TGFβ e PMN-elastase concentrations in EDTA plasma will be measured by multiplex assay using the Luminex 100™ system (Luminex) and custom Milliplex Map Kits (Millipore), following the manufacturer’s instructions. The data will be analyzed using Xponent 3.1 software.

All samples will be collected by the researchers and will be processed and stored by the molecular biology laboratory at IDPC so that the measurements mentioned above are made when the kits are available and the selection of participants has been completed.

### Statistical methods

#### Statistical methods for primary and secondary outcomes

The results will be analyzed using software SPSS 20 (SPSS Inc., Chicago, IL, USA) and the level of significance adopted will be *p* < 0.05. Before performing comparative tests of quantitative variables, it will be assessed whether the parameters follow a normal distribution by the Kolmogorov-Smirnov test. For parametric data, the Student *t* test will be used and for non-parametric data, the Mann-Whitney. For comparison over time, repeated measures ANOVA test will be used. To compare the frequencies of the qualitative variables, the chi-square test (*χ*^2^) or Fisher’s exact test will be performed.

#### Interim analyses

A safety committee will meet each time we complete 25% (25 patients) of the cases to analyze the provisional data, generating a report on the need or not to interrupt the study if there is a statistically significant difference between the groups (*p* < 0.05) related to mortality in 30 days, risk of anesthetic awareness, or other adverse events associated with the anesthetic-surgical procedure.

#### Methods for additional analyses

The analysis of the subgroups of this study will be based on the relationship between preoperative characteristics (e.g., diabetes, hypertension, dyslipidemia, smoking, and medications for continuous use), a reduction in the inflammatory response after 90 min of exposure to the sevoflurane anesthetic during CPB, intubation times, length of stay in the ICU, and length of stay.

#### Methods in analysis to handle protocol non-adherence and any statistical methods to handle missing data

There is no analysis method for non-adherence to the protocol.

#### Plans to give access to the full protocol, participant-level data, and statistical code

No later than 5 years after the collection of the 1-year post randomization interviews, we will deliver a completely deidentified data set to an appropriate data archive for sharing purposes.

### Oversight and monitoring

#### Composition of the coordinating center and trial steering committee

The coordinating center of the study is composed of 3 members (responsible researcher, advisor, and co-supervisor), whose role is in the design, review, and clinical and laboratory analysis of the study results. The coordinating center has monthly meetings.

In the meantime, the result management and judgment committee will be formed by members who are not participating in the study, both from the institution and from outside the Trial Steering Committee (TSC), who will analyze the results and point out possible failures and improvements. This committee has meetings every 2 weeks.

The other members will be responsible for the management and processing of clinical and laboratory data. And the principal investigator is solely responsible for recruiting and obtaining the consent of the study participants.

#### Composition of the data monitoring committee, its role and reporting structure

This data monitoring committee will be formed by 2 members participating in the study that make up the Molecular Biology Laboratory of Dante Pazzanese Institute of Cardiology and who should independently prepare periodic reports to the funding body (FAPESP), following the model of that body to report on the progress of the project.

#### Adverse event reporting and harms

Serious adverse events (SAEs) and damage from the intervention are considered: death or cardiopulmonary arrest during anesthetic induction, anesthetic awareness, and difficulty to control postoperative pain. All cases will be analyzed and notified to an independent committee that will recommend or not to interrupt the trial at any time if the insignificance or impairment of the intervention is proven.

#### Frequency and plans for auditing trial conduct

Auditors will verify adherence to required clinical trial procedures and will confirm accurate data collection according to the Good Clinical Practice (GCP) guidelines. Study monitoring and follow-up, from the initial setup to final reporting, will be fulfilled according to current National and International requirements.

#### Plans for communicating important protocol amendments to relevant parties

Any changes and amendments to the original protocol were notified to the Ethics Committee of Dante Pazzanese Institute of Cardiology.

#### Dissemination plans

The results will be published as soon as the study is completed, first in the central library of the University of São Paulo as a requirement for completing the doctoral title of the main author. Subsequently, it will be sent for worldwide scientific assessment with all data open to the public interested in the subject.

All named authors adhere to the authorship guidelines of Trials. All authors have agreed for publication.

## Discussion

Inflammatory cytokines such as PMN-elastase have different dynamics in their release after inflammatory leukocyte activation [12]. PMN-elastase is contained in polymorphonuclear granules and is released early, accounting for extracellular immune defense after exposure to the CPB circuit with a peak concentration within 90 min in vitro [2]. Interestingly, the inhibition of neutrophil activation as suggested in some experimental studies by the specific neutrophil inhibitor called sivelestat [13, 14] has reduced PMN-elastase levels in the same way as inhaled anesthetics [2].

Some studies have indicated that the adenosine triphosphate (ATP)-sensitive potassium channel may play a fundamental role in the protection of the myocardium induced by sevoflurane [15, 16]. There are clinical studies demonstrating that sevoflurane reduces neutrophil adhesion in the coronary system in a cardiac model of IRI [17], it inhibits neutrophil migration [18], the generation of oxygen free radicals by inflammatory cells [19], pulmonary complications [20], and also the release of cytokines by cultured human peripheral mononuclear cells [21]. These studies suggest that sevoflurane may protect against IRI by inhibition pro-inflammatory cytokines used during CPB, as opposed to a study using sevoflurane before CPB [22].

It is known that IL-8 can activate neutrophils. This provides an increase in neutrophil adhesion receptors such as Mac-1, which indirectly ends up harming the microcirculation and damaging the tissues around it. This response is maintained by a vicious cycle that can be suppressed by reducing the release of IL-8 [23, 24].

The release of IL-6 as well as IL-8 negatively impacts on myocardial contractility because it produces a downregulation of the beta-receptors [25] post-IR. Therefore, promoting a reduction of these two cytokines may result in better cardiac outcomes in the postoperative period.

In recent years, new evidence reinforces the importance of epigenetic studies, which involve DNA methylation, histone acetylation, and miRNA expression, especially the global expression profile of miRNAs (miRNome) in the search for new post-control mechanisms as well as their participation in the development of many diseases. Some research indicates that miRNAs have great potential as new molecular biomarkers of complex diseases, such as cancer and atherosclerosis, as well as susceptibility to infectious diseases [9, 10].

In view of the above described mediators involved in the inflammatory response to CPB, we are going to elucidate their role in relation to the use of sevoflurane during CPB and the possible impacts on the recovery CABG surgery.

### Trial status

Recruitment began on July 29, 2016, and ended on June 26, 2020. Consecutive participants who sign the written informed consent, aged 18 years or older, are enrolled. The study progress is updated monthly. The recruitment of patients in the study actually ended on June 26, 2020, when the last case selected following the inclusion and exclusion criteria was operated, completing 100 patients (50 in the sevo group and 50 in the control group). We have tried to submit the paper for many times before, but it took a long time with many comments and delays to be finally accepted for review (June 20, 2020).

So far, we still do not have the final results although the study is in the final phase of data analysis and laboratory testing with 130 patients being listed.

The authors are solely responsible for the design and conduct of this study, all study analyses and drafting and editing of the paper.

The results will be published as soon as the study is completed, first in the central library of the University of São Paulo as a requirement for completing the doctoral title of the main author. Subsequently, it will be sent for worldwide scientific assessment with all data open to the public interested in the subject. No later than 5 years after the collection of the 1-year post randomization interviews, we will deliver a completely deidentified data set to an appropriate data archive for sharing purposes.

This clinical trial has been approved to receive funding from the São Paulo State Research Support Foundation (FAPESP, acronym Portuguese), (reference 2017/21306-1).

## Supplementary Information

**Additional file 1:** Appendix 1.

## Data Availability

The datasets and their analyses will be available from the corresponding author during the trial by special request. All samples will be collected by the researchers and will be processed and stored by the molecular biology laboratory at IDPC so that the measurements mentioned above are made when the kits are available and the selection of participants has been completed.

## Abbreviations

CPB: Cardiac surgery with cardiopulmonary bypass
PMN: Polymorphonuclear
IL: Interleukins
TNFα: Alpha tumor necrosis factor
IRI: Ischemia reperfusion injury
miRNAs: MicroRNAs
ICU: Intensive care unit
CABG: Coronary artery bypass graft
COPD: Chronic obstructive pulmonary disease
BMI: Body mass index
SEVO: Sevoflurane
TIVA: Total intravenous anesthesia
MAC: Minimum alveolar concentration
TCI: Target-controlled infusion
MCI: Manually controlled infusion
mRNA: Messenger RNA
BIS: Bispectral index
MAP: Mean arterial pressure
TGFβ: Beta tumor growth factor
SD: Standard deviation
GAPDH: Glyceraldehyde 3-phosphate dehydrogenase
B2M: Beta-2-microglobulin
HPRT1: Hypoxanthine phosphoribosyltransferase
SDHA: Succinate dehydrogenase complex flavoprotein subunit A
UBC: Ubiquitin C
HMBS: Hydroxymethylbilane synthase
ATP: Adenosine triphosphate
