# Supplementary material for: Effect of sevoflurane on the inflammatory response during cardiopulmonary bypass in cardiac surgery: the study protocol for a randomized controlled trial
Source: Trials. 2021 Jan 6;22:25. doi: 10.1186/s13063-020-04809-x (PMC7789561; doi:10.1186/s13063-020-04809-x)
Supplement: Supplementary file 1 — Additional file 1: Appendix 1. [file 13063_2020_4809_MOESM1_ESM.docx]

**APPENDIX 1**

**Decompensated heart failure:** Defined by the Framingham criteria for heart failure and the New York Heart Association type IV classification.

**Decompensated hepatic failure:** Follows Child-Pugh's classification (types A, B and C).

**Acute kidney failure:** The AKI was defined as an increase greater than or equal to 50% over the baseline value and / or drop of greater than or equal to 25% of the glomerular filtration rate (GFR) and / or a decrease in diuresis of less than 0.5 ml / kg / h for 6 h or more. The "acute" element of the definition requires increased creatinine to occur within 7 days or less.

**Need for pacemaker:** Use of a temporary pacemaker to maintain cardiac output.

**Reoperation:** Surgical approach in the first 24 hours after the first intervention due to bleeding and or change in cardiologic clinical status with hemodynamic instability.

**Reintubation:** Need for mechanical ventilation after extubation.

**Arrhythmias:** Electrocardiographic changes in the 12-lead electrocardiogram performed at the Intensive Care Unit (ICU).

**Massive transfusion:** Infusion of blood products equivalent to 1.5 times the patient's blood volume or 20 units of red blood cell concentrate.

**Myocardial infarction:** Myocardial infarction confirmed by echocardiographic changes such as hypokinesia, dyskinesia or akinesia.

**Death:** Cardiac death in the first 24 hours.
